# What Can We Demonstrate?

**Published:** 1896-10

**Authors:** G. A. Mills

**Affiliations:** New York


### WHAT CAN WE DEMONSTRATE?

BY DR. G. A. MILLS, NEW YORK.

In the June number of the International Dental Journal, the writer, in criticising the omission of *practical* pathology in Professor Abbott's late book, adds, "Dental teaching to-day needs a work of this kind more than upon any other subject." The truth of this statement has been too apparent to need any comment. To prove this one has but to recall the fact of the multiplicity of methods of procedure in the department of the so-called "operative dentistry. What to do, and why do you do it? has had so little answer, it is no wonder that practice has so wofully wabbled in the past. In every department of life agnosticism is lifting her voice,—"How do you know?" Certainly, in the material world, demonstration is or can be made possible, and as truly so in the spiritual. In the material world the material eye can see what is demonstrated. Just as truly can the spiritual eye. How to arrive at these demonstrations is by a faithful following of teachings given in both departments. If a teacher cannot demonstrate his line of teaching we must recognize that he is unfitted for his part. Now, it is absurd to say that a line of uniformly successful dealing cannot be demonstrated. We use the word uniformly in an advisable sense. We do not hesitate to assert that all the lesions "*orally expressed by malnutrition*" (the *best* term that we have had in all this late *pyrotechnic* display covering the pages of our journals) are amenable to a (uniformly) successful correction, and *can be* demonstrated. There is no ambiguity in such a declaration; for if the one making it cannot prove it he falls.

Long experience ought to be a good teacher. It can be proved that the multitudinous methods that have been so elaborately presented to our calling are largely unnecessary; for simpler ones are only needed (and far fewer ones also). The best knowledge is always the simplest. In view of these statements, it is easy to assert that no "practical pathology" will be placed in our hands except by following a continued line of practice taught and demonstrated over the patient, showing the conditions *then* and *now*, and then, and only then, will the controversy cease. When you have taught and demonstrated, that settles it.

We have long held to the thought that the only intelligent way to produce a practical work of pathology for dental practitioners would be to give a continued series of surgical clinics before a class of students, such clinics being given twice a week for a term of say six months; have a complete record noted down by a stenographer and give a printed copy of same to each student; they, observing the teachings and the results, would be fitted to enter into practice as no other class of students have ever been. The value of such a course of teaching could hardly be computed. From this would come a class of practitioners that could demonstrate to the public the importance of our services. From this course of teaching could be produced work of success. We assert that it is possible to give such a series of teachings.

